# Patients with usual vulvar intraepithelial neoplasia-related vulvar cancer have an increased risk of cervical abnormalities

**DOI:** 10.1038/sj.bjc.6605124

**Published:** 2009-06-09

**Authors:** R P de Bie, H P van de Nieuwenhof, R L M Bekkers, W J G Melchers, A G Siebers, J Bulten, L F A G Massuger, J A de Hullu

**Affiliations:** 1 Department of Obstetrics and Gynaecology (791), Radboud University Nijmegen Medical Centre, P.O. Box 9101, 6500 HB Nijmegen, The Netherlands; 2 Department of Medical Microbiology (699), Radboud University Nijmegen Medical Centre, P.O. Box 9101, 6500 HB Nijmegen, The Netherlands; 3 Department of Pathology (826), Radboud University Nijmegen Medical Centre, P.O. Box 9101, 6500 HB Nijmegen, The Netherlands

**Keywords:** vulvar squamous cell carcinoma, usual vulvar intraepithelial neoplasia, cervical high-grade squamous intraepithelial lesion, human papillomavirus

## Abstract

**Background:**

Vulvar squamous cell carcinoma (SCC) originates the following two pathways, related to differentiated (d) vulvar intraepithelial neoplasia (VIN) or to human papillomavirus (HPV)-related usual (u) VIN. Multicentric HPV infections (cervix, vagina and vulva) are common. We hypothesise that patients with a uVIN-related vulvar SCC more often have cervical high-grade squamous intraepithelial lesions (HSILs) compared with women with dVIN-related vulvar SCC.

**Methods:**

All vulvar SCCs (201) were classified to be dVIN- (*n*=164) or uVIN related (*n*=37). Data with regard to the smear history and cervical histology were retrieved from PALGA, the nationwide Netherlands database of histo- and cytopathology. For HSIL cervical smears of which histology was taken, HPV DNA analysis on both the vulvar and cervical specimens was performed.

**Results:**

At least one smear was available in 145 (72%) of the 201 patients. Patients with a uVIN-related vulvar SCC more often had an HSIL compared with patients with a dVIN-related SCC (35 *vs* 2%, *P*<0.001). A total of 10 of the 13 HSILs were histologically assessed and identical HPV types were found in the vulva and cervix.

**Conclusion:**

These data emphasise the necessity to differentiate between dVIN- and uVIN-related vulvar tumours and to examine the entire lower female ano-genital tract once an uVIN-related lesion is found.

Vulvar squamous cell carcinoma (SCC) accounts for 3–5% of all gynaecological malignancies and originates following two separate pathways. One type of vulvar SCC, accounting for ∼80%, occurs mainly in older patients and is mostly of the keratinising type, associated with lichen sclerosus (LS) and/or differentiated vulvar intraepithelial neoplasia (dVIN) as premalignant lesions. The other type, accounting for ∼20%, is human papillomavirus (HPV) related, occurs in younger patients, and has usual VIN (uVIN) as premalignancy (van der Avoort *et al*, 2006). Recently, we found that uVIN-associated vulvar SCC patients had a significantly better disease-free survival than dVIN-associated vulvar SCC patients (van de Nieuwenhof *et al*, 2009b). The majority of uVIN-related vulvar SCCs are caused by HPVs 16, 18 and 33 (Toki *et al*, 1991; Hording *et al*, 1994; Iwasawa *et al*, 1997; Pinto *et al*, 2004; Hampl *et al*, 2006). The development of uVIN and vulvar SCC mirrors that of cervical intraepithelial neoplasia (CIN) and cervical SCC, the latter caused by (high-risk human papillomavirus) hrHPV in nearly 100% of the cases (Walboomers *et al*, 1999; Bekkers *et al*, 2004). A subdivision on the basis of hrHPV DNA presence between the two types of vulvar SCC does not separate the two different pathways accurately. High-risk HPV presence does not *per se* indicate a causal role in the carcinogenesis and may represent a non-significant or transient infection. A better method of subdividing the two types of vulvar SCC is on the basis of the histology of the adjacent VIN lesion (Vilmer *et al*, 1998; Rouzier *et al*, 2001; van de Nieuwenhof *et al*, 2009b).

Multicentric HPV infections affecting vulva, cervix, vagina and anus simultaneously have been described in several studies (Adamek *et al*, 2003; Bekkers *et al*, 2004; Lara-Torre and Perlman, 2004; Feng and Kiviat, 2005; Goffin *et al*, 2006; Hampl *et al*, 2007), making a thorough examination of the entire lower female ano-genital tract obligatory. A decreased immune response to hrHPV has been suggested to be the cause of these multicentric hrHPV infections (Hampl *et al*, 2007), although it is also possible that HPV affects cervix and vulva consecutively.

In this study, we investigate whether patients with uVIN-related vulvar SCCs more often have abnormal cervical smears and/or histologically confirmed cervical (pre)malignancies compared with women with dVIN/LS-related vulvar SCC. Moreover, we investigate which hrHPV types play a role in either uVIN-related vulvar SCCs and cervical (pre)malignancies.

## Patients and methods

Between 1988 and 2007, 209 consecutive patients were treated for their primary invasive or recurrent SCC of the vulva at the Radboud University Nijmegen Medical Centre, The Netherlands. Data were stored in a database. All histopathological slides were revised to classify the adjacent lesion as uVIN, LS and/or dVIN, to categorise the vulvar cancers into uVIN- or dVIN related (Park *et al*, 1991; Wilkinson, 1992; Carlson *et al*, 1998; Yang and Hart, 2000; Chiesa-Vottero *et al*, 2006). In eight cases, the subdivision was impossible because the diagnosis of the type of VIN lesion was uncertain because of massive intraepithelial inflammation, epithelial destruction due to massive ulceration or because the required tissue was not available. The remaining 201 patients constituted the study population.

### Cervical smears

Data with regard to the smear history and cervical histology were retrieved from PALGA, the nationwide Netherlands database of histo- and cytopathology. This database has had national coverage from 1991 onwards, showing all surgical specimens and cervical smears ever taken from each patient, both by the general practitioner and medical specialists (Casparie *et al*, 2007). The cervical histology and smear history were available for all the 201 patients.

Cervical smears were subdivided into smears taken more than 1 year before the diagnosis of vulvar SCC; smears taken within 1 year before the diagnosis of vulvar SCC; and smears taken after primary treatment for vulvar SCC.

In case multiple smears were taken during the study period, the most severe lesion was used for the analysis. Histology was preferred above cytology.

### Histology of adjacent VIN lesion

A subdivision on the basis of the histology of the adjacent VIN lesion (we adopted the new ISSVD Classification (Sideri *et al*, 2005)) was considered the most appropriate method to subdivide the vulvar SCCs into a pathway (van de Nieuwenhof *et al*, 2009b). Every original surgical specimen was revised for the presence of LS and/or VIN lesions, based on the current histopathological characteristics (Hart, 2001). If a patient had a high-grade squamous intraepithelial lesion (HSIL) resulting in a histological diagnosis, these specimens were retrieved in order to perform HPV DNA analysis on both the vulvar and cervical specimens. The presence of HPV was determined and genotyping was performed without the knowledge of patients' correlating tissues.

### SPF_10_-INNO LiPA HPV detection and genotyping

DNA was isolated from formalin-fixed paraffin-embedded tissue sections (6 *μ*m) with the EZ1 robot (with the DNA tissue kit of Qiagen Inc, Valencia, CA, USA) according to the standard procedures and used for PCR analysis. A negative water control was included with each batch of 10 samples. Broad-spectrum HPV DNA amplification was performed using a short PCR fragment assay (HPV SPF_10_ Line Blot 25, Labo Bio-medical products BV Rijswijk, The Netherlands). This assay amplifies a 65-bp fragment of the L1 open reading frame, and allows detection of a broad range of high-risk (hr), low-risk (lr) and possible hrHPV genotypes (Kleter *et al*, 1998; Melchers *et al*, 1999). Twenty-eight oligonucleotide probes that recognise 25 different types were tailed with poly(dT) and immobilised as parallel lines to membrane strips (Labo Bio-medical products BV Rijswijk, the Netherlands). The HPV genotypes detectable are hrHPVs 16, 18, 31, 33, 35, 39, 45, 51, 52, 56, 58, 59, and 68/73 and two probable hrHPV types (53 and 66), and lrHPV genotypes 6 and 11, and so on. The HPV genotyping assay was performed as described earlier (van Hamont *et al*, 2006). The line probe assay (LiPA) strips were visually inspected, and interpreted using the provided reference guide. As a quality control for the presence of DNA and absence of PCR inhibitors in the isolated material a *β*-globin PCR was performed, as described by Snijders *et al* (2006). For the real-time PCR method, 5 *μ*l of DNA was used.

### Statistical methods

The Kolmogorov–Smirnov test was used to evaluate whether the variables age, year of diagnosis, and first-line treatment of the two groups had a normal distribution. As the variables were not all normally distributed, all analyses were performed using non-parametrical tests. For analytical purposes, the histology and pap smears were subdivided into the Bethesda nomenclature, low-grade squamous intraepithelial lesions (LSILs) or less, and at least an HSIL. Fisher's exact test was used to compare hrHPV presence in uVIN and dVIN lesions adjacent to vulvar SCCs and to compare the different groups of vulvar SCC patients with respect to cervical abnormalities. *P*-values presented are two-tailed and the results were statistically significant at *P*<0.05.

## Results

The data of a total number of 201 patients were analysed. The median age of the patients at diagnosis was 71 years (range 30–92). The median age at diagnosis of the dVIN/LS-related pathway group (72 years, range 31–92) and the uVIN-related pathway group (54 years, range 30–88) differed significantly (*P*<0.001).

### Histology of the adjacent VIN lesion and HPV

Histopathological review of the VIN lesion directly adjacent to the tumour led to a total number of 37 (18%) tumours that were considered to follow the uVIN-related pathway, and 164 (82%) that were considered to follow the dVIN/LS-related pathway. Of the 164 dVIN/LS-related tumours, 54 (33%) tissues showed solitary dVIN, 22 (13%) tissues showed solitary LS and 88 (54%) tissues showed dVIN in combination with LS, as surrounding lesions. Of 168 tumours (32 uVIN and 136 dVIN/LS), both histology of the adjacent VIN lesion and HPV presence and typing could be performed. Of the uVIN-related tumours, 27 of the 32 (84.4%) were found hrHPV positive and 13 out of 136 (9.6%) of the dVIN/LS-related tumours were found hrHPV positive (Table 1). For the uVIN-related pathway, high-risk HPV types predominated, in particular types 16 (62%) and 33 (17%). The other 21% were caused by types 18, 52 and 58. Of the 13 hrHPV-positive tumours in the dVIN-related pathway, six tumours were found to be HPV 16 positive (46%) and the other seven were found to be HPV 18, 33, 52 or 53 positive.

### Smear histories

From 28% of the patients, no cervical smears or cervical biopsies had ever been taken. These patients had a median follow-up after their vulvar SCC of 8 years (range 1–20), without any clinical indication of cervical disease; 87% of these women were older than 55 years of age from the start of the national screening program for cervical cancer in 1988, as a result of which they exceeded the age limit to be invited. Of the 145 patients from whom at least one smear was available, the most severe lesion was taken: of these patients, 9.0% (*n*=13) had a smear indicating an HSIL. Patients with uVIN-related tumours had significantly more cervical smears indicating an HSIL, compared with patients with LS/dVIN-related tumours, 11 of 31 (35%) and 2 of 114 (2%) (*P*<0.001), respectively (Table 2).

### HSIL on cytology, histologically assessed

Histological specimens were examined after an HSIL smear in 10 of the 13 patients: in two cases there was a CIN I lesion, seven were CIN III lesions and one seemed to be an invasive cervical SCC. Chronologically, in five cases the CIN lesion was diagnosed before the vulvar SCC with an interval in time from 5 to 13 years (cases 2; 3; 7; 8; and 10); in four cases the CIN lesion was diagnosed simultaneously with the vulvar SCC (cases 1; 4; 6; and 9); and in one case the CIN lesion was diagnosed 6 years after the vulvar SCC (case 5).

Three patients with HSIL on cytology had, remarkably, no histological follow up. One patient died on account of a vulvar recurrence soon after the HSIL was diagnosed. One had a cytological follow up with normalisation of the smears in 5 years without interference of biopsies or excisions. The other particular patient was diagnosed with a vulvar SCC in 2004 and simultaneously had a HSIL on cervical cytology without further cervical follow up. A total of 9 of the 10 histologically confirmed tumours showed uVIN in the surrounding tissue and were HPV DNA positive. The one that showed dVIN in the surrounding tissue was HPV DNA negative. A cervical lesion of one patient showed negative *β*-globin after DNA extraction. In all tissues of the other eight patients, the same HPV types were found in both the vulvar and cervical specimens (Table 3).

## Discussion

Our study on a large group of patients with vulvar SCCs (201 cases), with regard to cervical (pre)malignancies, showed that 9% of all patients with available cytology of the cervix had a smear suggestive for a HSIL. Divided between the two pathways, 35% of the patients with uVIN-related vulvar SCCs had a smear suggestive for a HSIL, compared with only 2% of patients whose vulvar SCC was dVIN related (*P*<0.001). For uVIN-related SCCs, this is a more than 10-fold incidence compared with the overall incidence of a HSIL in The Netherlands (Vereniging Integrale Kankercentra, 2009). In 19% (*n*=7) of the total 37 patients with a uVIN-related vulvar SCC, a histologically proven cervical HSIL or SCC was found during a follow up of 20 years. Moreover, all of these uVIN-related vulvar SCCs and cervical lesions in the same patient showed the identical hrHPV type. These identical hrHPV types were seen in vulvar SCCs and cervical lesions even after an interval of more than 10 years. This may be one long-standing infection or a new infection with the exact same type of virus. Particularly as less common types were found as well, a long-standing infection is most likely. Furthermore, if a patient's immune system is not able to clear a hrHPV infection, that patient is thought to be more prone to develop multicentric (pre)malignancies. In most cases, the cervical lesion preceded the vulvar SCC, although vulvar SCCs preceding cervical lesions were seen as well. The difficulty is whether the date of diagnosis accurately reflects the origin of the lesions. It may well be possible that these lesions had initially been asymptomatic and present for a longer period before diagnosis. Our data suggest that the identical long-standing hrHPV infection is causing these multicentric (pre)malignancies. This emphasises the concept that a patient with a hrHPV-related ano-genital (pre)malignancy is at a higher risk to develop another hrHPV-related (pre)malignancy. We previously reported that 41% of uVIN patients had a past, simultaneous or future HPV-induced cervical, vaginal or anal lesion (van de Nieuwenhof *et al*, 2009a). Unfortunately, in this cohort of vulvar SCC patients, we were not able to retrieve data about other multicentric sites of the lower female ano-genital tract prone to hrHPV infection. This higher risk to develop other hrHPV-related (pre)malignancies obliges us to differentiate between dVIN and uVIN lesions followed by a precise examination of the entire lower female ano-genital tract in case of a vulvar carcinoma, especially in case of a tumour from the uVIN-related pathway.

In general, several studies showed that patients with uVIN-related vulvar SCC are younger than dVIN/LS-related vulvar SCC patients (Hording *et al*, 1994; Hampl *et al*, 2006; Hoevenaars *et al*, 2008; van de Nieuwenhof *et al*, 2009a). In our study, we confirm this observation with a significant difference in age distribution between the two different groups, 72 years for the hrHPV-unrelated and 54 years for the hrHPV-related groups (*P*<0.001). If we compare the median age for cervical SCC, which is caused by hrHPV in nearly 100% of the cases, with the median age for uVIN/hrHPV-related vulvar SCC, we find a comparable age, 51 years and 54 years, respectively (Hacker, 2000). This may suggest that a hrHPV infection brings about an earlier age of onset of uVIN-related vulvar carcinogenesis compared with the dVIN-related vulvar carcinogenesis. In this study, cervical (pre)malignancies with compatible hrHPV types were diagnosed as well before, simultaneous as after a uVIN-related vulvar SCC.

We found a hrHPV prevalence of 23.4% for vulvar SCC in general. The percentage of hrHPV DNA-positive vulvar SCC (23.4%) is slightly higher than the percentage of tumours with adjacent uVIN (18.4%). These findings confirm that not all hrHPV infections finally lead to vulvar SCC. As hrHPV often resolves spontaneously, most likely these five percent additional infections were transient infections, yet to be cleared.

Of the 201 patients, in 56 cases (28%) no cervical smear information was available: this may be because of the limitation in coverage of the PALGA database (national coverage from 1991 onwards) or because of the fact that these women never had a cervical smear taken. Worth mentioning is that 87% of these women exceeded the age limit to be invited for the national screening program for cervical cancer. Still, at diagnosis, in 105 of the 201 (52%) cases no cervical smear was taken around the diagnosis of the vulvar SCC. Approximately one-fifth of the vulvar SCCs is uVIN related, and simultaneous cervical and vulvar infections have previously been described (Adamek *et al*, 2003; Lara-Torre and Perlman, 2004; Feng and Kiviat, 2005; Goffin *et al*, 2006). Therefore, a cervical smear should always be taken during the diagnostic process of patients with a uVIN-related vulvar SCC. During the studied period (1988–2007), the role of hrHPV became more evident. In this study, for all patients diagnosed after 2002, a cervical smear was taken. This suggests that the cervical smear is now more embedded in the diagnostic routine of vulvar SCC. Our results show that it is important not to overlook or underestimate the possibility of a multicentric HPV infection, causing both cervical and vulvar (pre)malignancies. Although there is no evidence, probably because of the low number of patients that developed a HSIL lesion after vulvar SCC, we would suggest: all vulvar SCC patients have a cervical smear during the diagnostic process of their vulvar SCC; for uVIN-associated vulvar SCC patients a 2-yearly cervical smear should be performed during follow up.

In conclusion, patients with a uVIN-related vulvar SCC have an increased incidence of cervical (pre)malignancies with identical hrHPV types in both cervical and vulvar (pre)malignancies. This points to the multicentric development of (pre)malignancies and emphasises the necessity to differentiate between dVIN- and uVIN-related vulvar tumours. The examination of the entire lower female ano-genital tract in case of a hrHPV-related vulvar lesion should therefore be obligatory.

## Figures and Tables

**Table 1:** Distribution of high-risk human papillomavirus (hrHPV) in relation to the pathological pathway in 168 tissues of which both HPV DNA analysis and pathological pathway was available, *P*<0.001

	**hrHPV+**	**hrHPV−**	**Total**
uVIN	27	5	32
dVIN/LS	13	123	136
Total	40	128	168

dVIN=differentiated vulvar intraepithelial neoplasia; LS=lichen sclerosus; uVIN=usual vulvar intraepithelial neoplasia

**Table 2:** Total cervical specimens/smears of patients with vulvar squamous cell carcinoma, subdivided between two different pathways, related to LS/dVIN, or uVIN, *P*<0.001

	**LS, dVIN-related pathway**	**uVIN-related pathway**
⩽LSIL	112 (98%)	20 (65%)
⩾HSIL	2 (2%)	11 (35%)

dVIN=differentiated vulvar intraepithelial neoplasia; HSIL=high-grade squamous intraepithelial lesion; LS=lichen sclerosus; LSIL=low-grade squamous intraepithelial lesion; uVIN=usual vulvar intraepithelial neoplasia.

**Table 3:** Ten cases of vulvar SCCs and cytologically a high-grade squamous intraepithelial lesion of the cervix with histological correlating lesion and HPV types

**Case**	**Adjacent lesion**	**Year**	**Age**	**Type of tumour**	**HPV**
1	uVIN	2007	65	Vulvar SCC	**16**
		2007	65	Cervical SCC	**16**
2	uVIN	1990	34	VIN III	**33**
		1990	34	Cervical carcinoma *in situ*	**33**
	uVIN	2003	47	Vulvar SCC	**33**
3		1990	38	CIN III	**16**
	uVIN	2003	51	Vulvar SCC	**16**
4	uVIN	1998	37	Vulvar SCC	**16**
		1998	37	CIN III	**16**
	uVIN	2006	45	Vulvar SCC	**16**
5	uVIN	1999	45	Vulvar SCC	16,**52**
		2005	51	CIN III	**52**
6	uVIN	2004	37	Vulvar SCC	**16**,18,54
		2004	37	CIN III	**16**
		2007	40	CIN III	**16**
7		1986	37	CIN III	**58**
	uVIN	1992	43	Vulvar SCC	**58**
8		1997	48	CIN I	**16**
	uVIN	2002	53	Vulvar SCC	**16**,58
9	uVIN	2004	58	Vulvar SCC	**16**
		2004	58	CIN I	*β*-Globin negative
10		1994	49	CIN III	31
	LS+dVIN	2005	60	Vulvar SCC	Negative

CIN=cervical intraepithelial neoplasia; dVIN=differentiated vulvar intraepithelial neoplasia; HPV=human papillomavirus; LS=lichen sclerosus; SCCs=squamous cell carcinomas; uVIN=usual vulvar intraepithelial neoplasia. Bold values indicate identical hrHPV types.
